# Current state of knowledge of hepatic encephalopathy (part III): non-absorbable disaccharides

**DOI:** 10.1007/s11011-016-9910-2

**Published:** 2016-09-16

**Authors:** Marsha Y. Morgan

**Affiliations:** UCL Institute for Liver & Digestive Health, Division of Medicine, Royal Free Campus, University College London, London, NW3 2PF UK

**Keywords:** Hepatic encephalopathy, lactulose, lactitol, efficacy, safety

## Abstract

Nonabsorbable disaccharides have been the mainstay of treatment for hepatic encephalopathy since introduced into clinical practice in 1966. Their beneficial effects reflect their ability to reduce the intestinal production/absorption of ammonia. A recent Cochrane review confirmed the efficacy and safety of nonabsorbable disaccharides for the treatment and prevention of hepatic encephalopathy in patients with cirrhosis. The findings were robust and support the use of nonabsorbable disaccharides as a first line treatment for hepatic encephalopathy, in this patient population, and for its prevention.

The non-absorbable disaccharide, lactulose, was first introduced into clinical practice, for the treatment of hepatic encephalopathy, in 1966. The second generation non-absorbable disaccharide, lactitol, was introduced in the mid-1980s. These non-absorbable disaccharides have become the mainstay of treatment for this condition.

These synthetic disaccharides are broadly classified as *osmotic laxatives* but have also been classified as *prebiotics,* a generic term referring to agents that induce the growth and/or activity of commensal micro-organisms. They are also often referred to as *functional foods* placing them conceptually intermediate between foods and drugs.

In this review the mechanism of action of the non-absorbable disaccharides will be reviewed; their clinical efficacy and safety for the treatment of hepatic encephalopathy will be examined and the barriers to their use, in this context, explored.

## Mechanism of action

The human small intestinal mucosa does not possess enzymes capable of splitting these synthetic disaccharides into their consistent parts. Thus, they are not processed or absorbed in the small intestine but pass unchanged into the large intestine. There they are extensively metabolized by colonic bacteria to their constituent monosaccharides and then to volatile fatty acids and hydrogen. Their beneficial effects reflect their ability to reduce the intestinal production/absorption of ammonia, which is achieved in four ways:

(i) *A laxative effect*: the colonic metabolism of the non-absorbable disaccharides results in an increase in intraluminal gas formation, an increase in intraluminal osmolality, a reduction in intraluminal pH, and an overall decrease in transit time;
(ii) *Bacterial uptake of ammonia*: the intraluminal changes in pH result in a leaching of ammonia from the circulation into the colon. The colonic bacteria use the released volatile fatty acids as substrate and proliferate. In doing so, they use the trapped colonic ammonia as a nitrogen source for protein synthesis. The increase in bacterial numbers additionally ‘bulks’ the stool and contributes to the cathartic effect (Weber et al. 1987);
(iii) *Reduction of intestinal ammonia production*: nonabsorbable disaccharides inhibit glutaminase activity and interfere with the intestinal uptake of glutamine and its subsequent metabolism to ammonia (van Leeuwen et al. 1988);
(iv) *Beneficial effects on the gut microbiome:* cirrhosis is associated with dysbiosis and changes to the colonic mucosal microbiome (Qin et al. 2014); there is also evidence of further changes in the gut microbiome in patients with hepatic encephalopathy (Bajaj et al. 2012). Non-absorbable disaccharides can beneficially affect microbiota composition (Riggio et al. 1990; Bajaj et al. 2012).

## Clinical efficacy

A Cochrane review, published in 2004, found insufficient evidence to recommend the use of non-absorbable disaccharides for the treatment of hepatic encephalopathy in patients with cirrhosis (Als-Nielsen et al. 2004). However, there were a number of methodological issues with this review including: the selection of the included trials; the reporting of bias domains; and the lack of statistical power-all of which weakened the strength of the conclusions.

In 2014, the European and American Associations for the Study of the Liver (EASL/AASLD) published a joint practice guideline in which they recommended lactulose as the treatment of choice for overt hepatic encephalopathy and for secondary prevention after an index event (Vilstrup et al. 2014). They did not recommend routine treatment for minimal hepatic encephalopathy but stated that exceptions could be made, on a case-by-case basis, if driving skills, work performance, quality of life or cognitive function were impaired. They did not recommend primary prophylaxis for the prevention of hepatic encephalopathy except in patients ‘known to be at high risk’ which was not otherwise defined. The guideline mentions that lactitol is preferred in some centres but did not comment on the relative efficacy and safety of the two agents.

The authors of the EASL/AASLD guideline based their recommendations on clinical experience and on a formal review and analysis of recently published literature selecting studies for inclusion based on the appropriateness of the study design, a relevant number of participants and confidence in the participating centre and investigators. There is clearly a potential risk of bias in this approach.

**Table 1:** Beneficial effects of non-absorbable disaccharides on mortality in randomized clinical trials, by type of hepatic encephalopathy

Type of hepatic encephalopathy	RR (95 % CI)	Trials (n)	Patients (n)
Overt	0.36 (0.14–0.94)	6	172
Minimal	0.82 (0.24–2.86)	12	647
Prevention	0.63 (0.40–0.98)	6	668
Overall	0.59 (0.40–0.87)	24	1487

(adapted from Gluud et al. 2016)

The apparent discrepant views provided by the original Cochrane review (Als-Nielsen et al. 2004) and the latest EASL/AASLD practice guideline (Vilstrup et al. 2014) prompted a further review, under the Cochrane banner, of the role of non-absorbable disaccharides in patients with cirrhosis and hepatic encephalopathy (Gluud et al. 2016). A total of 38 randomized clinical trials involving 1828 participants were included and the analyses provided moderate quality evidence that use of non-absorbable disaccharides is associated with beneficial effects on hepatic encephalopathy, mortality, and serious adverse events when used to treat overt hepatic encephalopathy, minimal hepatic encephalopathy and to prevent hepatic encephalopathy. Lactulose and lactitol were equally as effective. More specifically the review showed:

### Hepatic encephalopathy

**Fig. 1:**
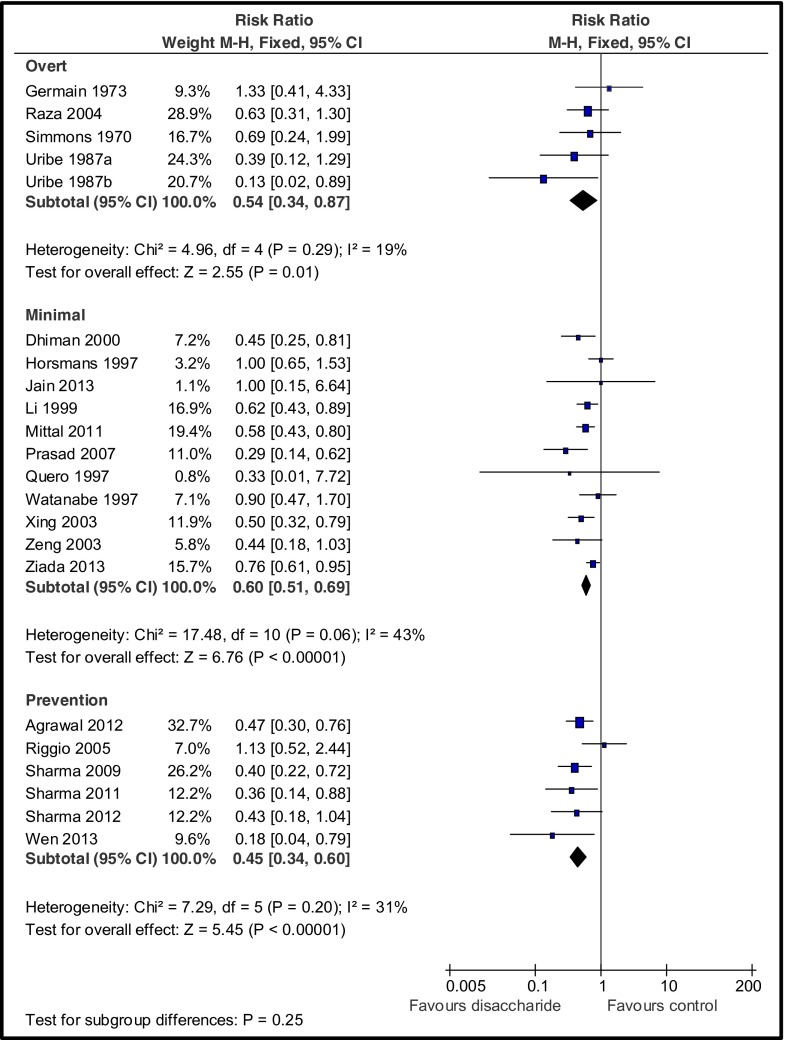
Beneficial effects of non-absorbable disaccharides on hepatic encephalopathy in randomized clinical trials against placebo/no intervention, by type of encephalopathy (adapted from Gluud et al. 2016)

Treatment with non-absorbable disaccharides was associated with a significant beneficial effect on hepatic encephalopathy with a number needed to treat (NNT) of six (Fig. 1).

### Mortality

Treatment was associated with a significant beneficial effect on survival with a NNT of 19; (Table 1).

**Table 2:** Beneficial effects of non-absorbable disaccharides on serious adverse events in randomized clinical trials

Serious adverse events	RR (95 % CI)	Trials (n)	Patients (n)
Liver failure	0.35 (0.11–1.15)	4	241
Spontaneous bacterial peritonitis	0.86 (0.32–2.30)	2	278
Variceal haemorrhage	0.46 (0.28–0.98)	9	681
Hepatorenal syndrome	0.47 (0.21–1.04)	3	278
Overall	0.47 (0.36–0.60)	24	1487

(adapted from Gluud et al. 2016)

### Serious adverse events

The risk of serious adverse events, including: liver failure, serious infections, spontaneous bacterial peritonitis, variceal haemorrhage and hepatorenal syndrome was significantly reduced with a NNT of six (Table 2):

The findings of this updated review (Gluud et al. 2016) contrast with those of the previous Cochrane review mainly because of the increased number of trials now available for review and the overall quality of the evidence. The findings endorse the EASL/AASLD guideline with regard to the treatment of overt hepatic encephalopathy and the prevention of recurrence after an index event but found additional evidence to support the treatment of minimal hepatic encephalopathy and for primary prophylaxis.

## Barriers to treatment

The non-absorbable disaccharides have a laxative effect and their use can be associated with nausea, bloating, diarrhoea and flatulence. This is more of a problem with lactulose syrup, which is contaminated with other sugars, than it is with the crystalline lactulose preparation or with lactitol. Adjusting the dosage to produce two semi-soft stools a day will optimize the beneficial effects whilst minimizing the side-effects. Many patients adapt well to long-term use of these agents seemingly developing tolerance to the side-effects over time; others, however, do not.

There is very little information on long-term compliance with treatment but little doubt that non-adherence is a major factor in ‘treatment failure’ (Bajaj et al. 2010; Volk et al. 2012). There are, however, factors other than side-effects that can affect compliance, including: (i) on the patients’ part: a lack of awareness of the need for long-term treatment; an inability to effectively titrate the treatment dosage and the inconvenience of treatment when away from home; and, (ii) on the physicians’ part: a failure to explain the multiple ways in which non-absorbable disaccharides produces their beneficial effects; an undue focus on the need to pass two semi-soft stools/day often resulting in the belief by the patient that as long as this is achieved there is no real need to take the medication; and the assumption that patients will comply with treatment and hence a failure to continuously check adherence.

## Outstanding issues

Non-absorbable disaccharides are an effective and safe treatment for hepatic encephalopathy in patients with cirrhosis. However, more trials, undertaken to rigorous standards and with clinically meaningful outcomes, are needed to inform decision making. In particular long-term trials are needed to assess the cost-benefit and cost-effectiveness of treating minimal hepatic encephalopathy and for primary prevention of hepatic encephalopathy.

More information on long term-compliance with treatment, outside of clinical trials, is needed and the reasons for non-adherence determined. This information should then be used to inform educational and support systems for patients, their caregivers and physicians to optimize treatment adherence and hence benefit.
